# Supplementary figures and images for: Cofilin-induced unidirectional cooperative conformational changes in actin filaments revealed by high-speed atomic force microscopy
Source: eLife. 2015 Feb 2;4:e04806. doi: 10.7554/eLife.04806 (PMC4337605; doi:10.7554/eLife.04806)

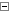

Supplement: Source code 1. — The Kodec4.4.7.39 for HS-AFM image viewing and analysis software is coded in Visual C# (Visual Studio 2010, Microsoft, USA). DOI: http://dx.doi.org/10.7554/eLife.04806.031 [file elife04806s001.zip › Kodec 4.4.7.39/_UpgradeReport_Files/UpgradeReport_Minus.gif]

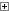

Supplement: Source code 1. — The Kodec4.4.7.39 for HS-AFM image viewing and analysis software is coded in Visual C# (Visual Studio 2010, Microsoft, USA). DOI: http://dx.doi.org/10.7554/eLife.04806.031 [file elife04806s001.zip › Kodec 4.4.7.39/_UpgradeReport_Files/UpgradeReport_Plus.gif]

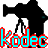

Supplement: Source code 1. — The Kodec4.4.7.39 for HS-AFM image viewing and analysis software is coded in Visual C# (Visual Studio 2010, Microsoft, USA). DOI: http://dx.doi.org/10.7554/eLife.04806.031 [file elife04806s001.zip › Kodec 4.4.7.39/Kodec/KodecIcon-002.png]

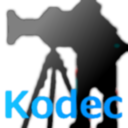

Supplement: Source code 1. — The Kodec4.4.7.39 for HS-AFM image viewing and analysis software is coded in Visual C# (Visual Studio 2010, Microsoft, USA). DOI: http://dx.doi.org/10.7554/eLife.04806.031 [file elife04806s001.zip › Kodec 4.4.7.39/Kodec/KodecIcon128.png]

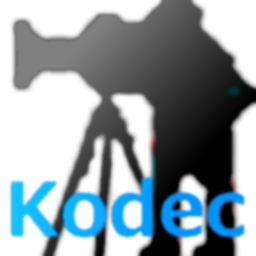

Supplement: Source code 1. — The Kodec4.4.7.39 for HS-AFM image viewing and analysis software is coded in Visual C# (Visual Studio 2010, Microsoft, USA). DOI: http://dx.doi.org/10.7554/eLife.04806.031 [file elife04806s001.zip › Kodec 4.4.7.39/Kodec/KodecIcon256.png]

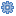

Supplement: Source code 1. — The Kodec4.4.7.39 for HS-AFM image viewing and analysis software is coded in Visual C# (Visual Studio 2010, Microsoft, USA). DOI: http://dx.doi.org/10.7554/eLife.04806.031 [file elife04806s001.zip › Kodec 4.4.7.39/Kodec/LocalResource/applications-system.png]

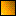

Supplement: Source code 1. — The Kodec4.4.7.39 for HS-AFM image viewing and analysis software is coded in Visual C# (Visual Studio 2010, Microsoft, USA). DOI: http://dx.doi.org/10.7554/eLife.04806.031 [file elife04806s001.zip › Kodec 4.4.7.39/Kodec/LocalResource/AutoTilt.bmp]

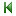

Supplement: Source code 1. — The Kodec4.4.7.39 for HS-AFM image viewing and analysis software is coded in Visual C# (Visual Studio 2010, Microsoft, USA). DOI: http://dx.doi.org/10.7554/eLife.04806.031 [file elife04806s001.zip › Kodec 4.4.7.39/Kodec/LocalResource/BackToTop.png]

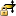

Supplement: Source code 1. — The Kodec4.4.7.39 for HS-AFM image viewing and analysis software is coded in Visual C# (Visual Studio 2010, Microsoft, USA). DOI: http://dx.doi.org/10.7554/eLife.04806.031 [file elife04806s001.zip › Kodec 4.4.7.39/Kodec/LocalResource/BaseLock.png]

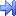

Supplement: Source code 1. — The Kodec4.4.7.39 for HS-AFM image viewing and analysis software is coded in Visual C# (Visual Studio 2010, Microsoft, USA). DOI: http://dx.doi.org/10.7554/eLife.04806.031 [file elife04806s001.zip › Kodec 4.4.7.39/Kodec/LocalResource/ClipEnd.png]

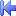

Supplement: Source code 1. — The Kodec4.4.7.39 for HS-AFM image viewing and analysis software is coded in Visual C# (Visual Studio 2010, Microsoft, USA). DOI: http://dx.doi.org/10.7554/eLife.04806.031 [file elife04806s001.zip › Kodec 4.4.7.39/Kodec/LocalResource/ClipStart.png]

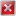

Supplement: Source code 1. — The Kodec4.4.7.39 for HS-AFM image viewing and analysis software is coded in Visual C# (Visual Studio 2010, Microsoft, USA). DOI: http://dx.doi.org/10.7554/eLife.04806.031 [file elife04806s001.zip › Kodec 4.4.7.39/Kodec/LocalResource/close_sq-010.png]

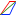

Supplement: Source code 1. — The Kodec4.4.7.39 for HS-AFM image viewing and analysis software is coded in Visual C# (Visual Studio 2010, Microsoft, USA). DOI: http://dx.doi.org/10.7554/eLife.04806.031 [file elife04806s001.zip › Kodec 4.4.7.39/Kodec/LocalResource/ColorMap.png]

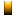

Supplement: Source code 1. — The Kodec4.4.7.39 for HS-AFM image viewing and analysis software is coded in Visual C# (Visual Studio 2010, Microsoft, USA). DOI: http://dx.doi.org/10.7554/eLife.04806.031 [file elife04806s001.zip › Kodec 4.4.7.39/Kodec/LocalResource/ColorMap_Bar.png]

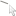

Supplement: Source code 1. — The Kodec4.4.7.39 for HS-AFM image viewing and analysis software is coded in Visual C# (Visual Studio 2010, Microsoft, USA). DOI: http://dx.doi.org/10.7554/eLife.04806.031 [file elife04806s001.zip › Kodec 4.4.7.39/Kodec/LocalResource/cs.png]

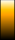

Supplement: Source code 1. — The Kodec4.4.7.39 for HS-AFM image viewing and analysis software is coded in Visual C# (Visual Studio 2010, Microsoft, USA). DOI: http://dx.doi.org/10.7554/eLife.04806.031 [file elife04806s001.zip › Kodec 4.4.7.39/Kodec/LocalResource/default.png]

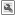

Supplement: Source code 1. — The Kodec4.4.7.39 for HS-AFM image viewing and analysis software is coded in Visual C# (Visual Studio 2010, Microsoft, USA). DOI: http://dx.doi.org/10.7554/eLife.04806.031 [file elife04806s001.zip › Kodec 4.4.7.39/Kodec/LocalResource/document-properties.png]

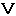

Supplement: Source code 1. — The Kodec4.4.7.39 for HS-AFM image viewing and analysis software is coded in Visual C# (Visual Studio 2010, Microsoft, USA). DOI: http://dx.doi.org/10.7554/eLife.04806.031 [file elife04806s001.zip › Kodec 4.4.7.39/Kodec/LocalResource/eracer - âRâsü[.png]

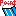

Supplement: Source code 1. — The Kodec4.4.7.39 for HS-AFM image viewing and analysis software is coded in Visual C# (Visual Studio 2010, Microsoft, USA). DOI: http://dx.doi.org/10.7554/eLife.04806.031 [file elife04806s001.zip › Kodec 4.4.7.39/Kodec/LocalResource/eracer(point).png]

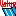

Supplement: Source code 1. — The Kodec4.4.7.39 for HS-AFM image viewing and analysis software is coded in Visual C# (Visual Studio 2010, Microsoft, USA). DOI: http://dx.doi.org/10.7554/eLife.04806.031 [file elife04806s001.zip › Kodec 4.4.7.39/Kodec/LocalResource/eracer(Value).png]

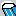

Supplement: Source code 1. — The Kodec4.4.7.39 for HS-AFM image viewing and analysis software is coded in Visual C# (Visual Studio 2010, Microsoft, USA). DOI: http://dx.doi.org/10.7554/eLife.04806.031 [file elife04806s001.zip › Kodec 4.4.7.39/Kodec/LocalResource/eracer.png]

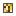

Supplement: Source code 1. — The Kodec4.4.7.39 for HS-AFM image viewing and analysis software is coded in Visual C# (Visual Studio 2010, Microsoft, USA). DOI: http://dx.doi.org/10.7554/eLife.04806.031 [file elife04806s001.zip › Kodec 4.4.7.39/Kodec/LocalResource/Filter.png]

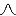

Supplement: Source code 1. — The Kodec4.4.7.39 for HS-AFM image viewing and analysis software is coded in Visual C# (Visual Studio 2010, Microsoft, USA). DOI: http://dx.doi.org/10.7554/eLife.04806.031 [file elife04806s001.zip › Kodec 4.4.7.39/Kodec/LocalResource/Gauss.png]

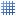

Supplement: Source code 1. — The Kodec4.4.7.39 for HS-AFM image viewing and analysis software is coded in Visual C# (Visual Studio 2010, Microsoft, USA). DOI: http://dx.doi.org/10.7554/eLife.04806.031 [file elife04806s001.zip › Kodec 4.4.7.39/Kodec/LocalResource/Grid.png]

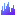

Supplement: Source code 1. — The Kodec4.4.7.39 for HS-AFM image viewing and analysis software is coded in Visual C# (Visual Studio 2010, Microsoft, USA). DOI: http://dx.doi.org/10.7554/eLife.04806.031 [file elife04806s001.zip › Kodec 4.4.7.39/Kodec/LocalResource/Histogram.png]

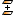

Supplement: Source code 1. — The Kodec4.4.7.39 for HS-AFM image viewing and analysis software is coded in Visual C# (Visual Studio 2010, Microsoft, USA). DOI: http://dx.doi.org/10.7554/eLife.04806.031 [file elife04806s001.zip › Kodec 4.4.7.39/Kodec/LocalResource/Integration.png]

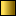

Supplement: Source code 1. — The Kodec4.4.7.39 for HS-AFM image viewing and analysis software is coded in Visual C# (Visual Studio 2010, Microsoft, USA). DOI: http://dx.doi.org/10.7554/eLife.04806.031 [file elife04806s001.zip › Kodec 4.4.7.39/Kodec/LocalResource/LineTilt.png]

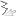

Supplement: Source code 1. — The Kodec4.4.7.39 for HS-AFM image viewing and analysis software is coded in Visual C# (Visual Studio 2010, Microsoft, USA). DOI: http://dx.doi.org/10.7554/eLife.04806.031 [file elife04806s001.zip › Kodec 4.4.7.39/Kodec/LocalResource/LM.png]

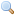

Supplement: Source code 1. — The Kodec4.4.7.39 for HS-AFM image viewing and analysis software is coded in Visual C# (Visual Studio 2010, Microsoft, USA). DOI: http://dx.doi.org/10.7554/eLife.04806.031 [file elife04806s001.zip › Kodec 4.4.7.39/Kodec/LocalResource/magnifier.png]

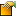

Supplement: Source code 1. — The Kodec4.4.7.39 for HS-AFM image viewing and analysis software is coded in Visual C# (Visual Studio 2010, Microsoft, USA). DOI: http://dx.doi.org/10.7554/eLife.04806.031 [file elife04806s001.zip › Kodec 4.4.7.39/Kodec/LocalResource/ManualTilt.png]

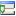

Supplement: Source code 1. — The Kodec4.4.7.39 for HS-AFM image viewing and analysis software is coded in Visual C# (Visual Studio 2010, Microsoft, USA). DOI: http://dx.doi.org/10.7554/eLife.04806.031 [file elife04806s001.zip › Kodec 4.4.7.39/Kodec/LocalResource/ManualTiltDialog.png]

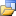

Supplement: Source code 1. — The Kodec4.4.7.39 for HS-AFM image viewing and analysis software is coded in Visual C# (Visual Studio 2010, Microsoft, USA). DOI: http://dx.doi.org/10.7554/eLife.04806.031 [file elife04806s001.zip › Kodec 4.4.7.39/Kodec/LocalResource/OpenFile.png]

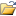

Supplement: Source code 1. — The Kodec4.4.7.39 for HS-AFM image viewing and analysis software is coded in Visual C# (Visual Studio 2010, Microsoft, USA). DOI: http://dx.doi.org/10.7554/eLife.04806.031 [file elife04806s001.zip › Kodec 4.4.7.39/Kodec/LocalResource/OpenFolder.png]

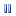

Supplement: Source code 1. — The Kodec4.4.7.39 for HS-AFM image viewing and analysis software is coded in Visual C# (Visual Studio 2010, Microsoft, USA). DOI: http://dx.doi.org/10.7554/eLife.04806.031 [file elife04806s001.zip › Kodec 4.4.7.39/Kodec/LocalResource/PauseHS.png]

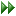

Supplement: Source code 1. — The Kodec4.4.7.39 for HS-AFM image viewing and analysis software is coded in Visual C# (Visual Studio 2010, Microsoft, USA). DOI: http://dx.doi.org/10.7554/eLife.04806.031 [file elife04806s001.zip › Kodec 4.4.7.39/Kodec/LocalResource/PlayFastHS.PNG]

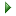

Supplement: Source code 1. — The Kodec4.4.7.39 for HS-AFM image viewing and analysis software is coded in Visual C# (Visual Studio 2010, Microsoft, USA). DOI: http://dx.doi.org/10.7554/eLife.04806.031 [file elife04806s001.zip › Kodec 4.4.7.39/Kodec/LocalResource/PlayHS.png]

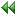

Supplement: Source code 1. — The Kodec4.4.7.39 for HS-AFM image viewing and analysis software is coded in Visual C# (Visual Studio 2010, Microsoft, USA). DOI: http://dx.doi.org/10.7554/eLife.04806.031 [file elife04806s001.zip › Kodec 4.4.7.39/Kodec/LocalResource/PlaySlowHS.png]

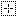

Supplement: Source code 1. — The Kodec4.4.7.39 for HS-AFM image viewing and analysis software is coded in Visual C# (Visual Studio 2010, Microsoft, USA). DOI: http://dx.doi.org/10.7554/eLife.04806.031 [file elife04806s001.zip › Kodec 4.4.7.39/Kodec/LocalResource/Point.png]

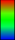

Supplement: Source code 1. — The Kodec4.4.7.39 for HS-AFM image viewing and analysis software is coded in Visual C# (Visual Studio 2010, Microsoft, USA). DOI: http://dx.doi.org/10.7554/eLife.04806.031 [file elife04806s001.zip › Kodec 4.4.7.39/Kodec/LocalResource/rainbow.png]

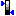

Supplement: Source code 1. — The Kodec4.4.7.39 for HS-AFM image viewing and analysis software is coded in Visual C# (Visual Studio 2010, Microsoft, USA). DOI: http://dx.doi.org/10.7554/eLife.04806.031 [file elife04806s001.zip › Kodec 4.4.7.39/Kodec/LocalResource/Rescale.png]

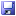

Supplement: Source code 1. — The Kodec4.4.7.39 for HS-AFM image viewing and analysis software is coded in Visual C# (Visual Studio 2010, Microsoft, USA). DOI: http://dx.doi.org/10.7554/eLife.04806.031 [file elife04806s001.zip › Kodec 4.4.7.39/Kodec/LocalResource/Save.png]

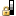

Supplement: Source code 1. — The Kodec4.4.7.39 for HS-AFM image viewing and analysis software is coded in Visual C# (Visual Studio 2010, Microsoft, USA). DOI: http://dx.doi.org/10.7554/eLife.04806.031 [file elife04806s001.zip › Kodec 4.4.7.39/Kodec/LocalResource/scaleLock.png]

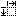

Supplement: Source code 1. — The Kodec4.4.7.39 for HS-AFM image viewing and analysis software is coded in Visual C# (Visual Studio 2010, Microsoft, USA). DOI: http://dx.doi.org/10.7554/eLife.04806.031 [file elife04806s001.zip › Kodec 4.4.7.39/Kodec/LocalResource/Size.png]

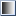

Supplement: Source code 1. — The Kodec4.4.7.39 for HS-AFM image viewing and analysis software is coded in Visual C# (Visual Studio 2010, Microsoft, USA). DOI: http://dx.doi.org/10.7554/eLife.04806.031 [file elife04806s001.zip › Kodec 4.4.7.39/Kodec/LocalResource/Tilt.png]

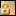

Supplement: Source code 1. — The Kodec4.4.7.39 for HS-AFM image viewing and analysis software is coded in Visual C# (Visual Studio 2010, Microsoft, USA). DOI: http://dx.doi.org/10.7554/eLife.04806.031 [file elife04806s001.zip › Kodec 4.4.7.39/Kodec/LocalResource/TiltLock.png]

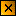

Supplement: Source code 1. — The Kodec4.4.7.39 for HS-AFM image viewing and analysis software is coded in Visual C# (Visual Studio 2010, Microsoft, USA). DOI: http://dx.doi.org/10.7554/eLife.04806.031 [file elife04806s001.zip › Kodec 4.4.7.39/Kodec/LocalResource/TiltOFF.bmp]

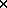

Supplement: Source code 1. — The Kodec4.4.7.39 for HS-AFM image viewing and analysis software is coded in Visual C# (Visual Studio 2010, Microsoft, USA). DOI: http://dx.doi.org/10.7554/eLife.04806.031 [file elife04806s001.zip › Kodec 4.4.7.39/Kodec/LocalResource/X.png]

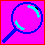

Supplement: Source code 1. — The Kodec4.4.7.39 for HS-AFM image viewing and analysis software is coded in Visual C# (Visual Studio 2010, Microsoft, USA). DOI: http://dx.doi.org/10.7554/eLife.04806.031 [file elife04806s001.zip › Kodec 4.4.7.39/Kodec/LocalResource/Zoom.bmp]

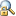

Supplement: Source code 1. — The Kodec4.4.7.39 for HS-AFM image viewing and analysis software is coded in Visual C# (Visual Studio 2010, Microsoft, USA). DOI: http://dx.doi.org/10.7554/eLife.04806.031 [file elife04806s001.zip › Kodec 4.4.7.39/Kodec/LocalResource/ZoomLock.png]
